# Apelin-13 alleviates contrast-induced acute kidney injury by inhibiting endoplasmic reticulum stress

**DOI:** 10.1080/0886022X.2023.2179852

**Published:** 2023-02-22

**Authors:** Qian Liu, Shao-Bin Duan, Lin Wang, Xiao-Qin Luo, Hong-Shen Wang, Ying-Hao Deng, Xi Wu, Ting Wu, Ping Yan and, Yi-Xin Kang

**Affiliations:** Department of Nephrology, Hunan Key Laboratory of Kidney Disease and Blood Purification, The Second Xiangya Hospital of Central South University, Changsha 410011, China

**Keywords:** Acute kidney injury, contrast media, apelin-13, endoplasmic reticulum stress, reactive oxygen species

## Abstract

Contrast-induced acute kidney injury (CI-AKI) is a severe complication associated with significant morbidity and mortality, and effective therapeutic strategies are still lacking. Apelin is an endogenous physiological regulator with antioxidative, anti-inflammatory and antiapoptotic properties. However, the role of apelin-13 in CI-AKI remains unclear. In our study, we found that the protein expression levels of apelin were significantly downregulated in rat kidney tissues and HK-2 cells during contrast media treatment. Moreover, we explored the protective effect of apelin-13 on renal tubule damage using *in vitro* and *in vivo* models of CI-AKI. Exogenous apelin-13 ameliorated endoplasmic reticulum stress, reactive oxygen species and apoptosis protein expression in contrast media-treated cells and rat kidney tissues. Mechanistically, the downregulation of endoplasmic reticulum stress contributed critically to the antiapoptotic effect of apelin-13. Collectively, our findings reveal the inherent mechanisms by which apelin-13 regulates CI-AKI and provide a prospective target for the prevention of CI-AKI.

## Graphical Abstract

**Figure:**
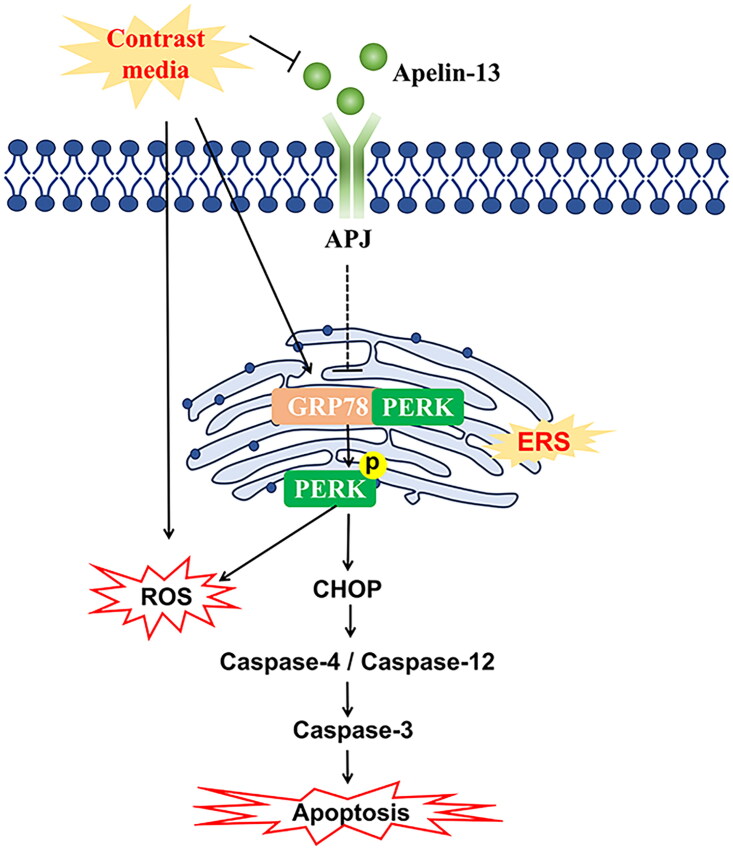

## 1. Introduction

Contrast-induced acute kidney injury (CI-AKI) is a frequent and serious complication of radiologic diagnosis and therapy using intravascular contrast media (CM) [1,2]. CI-AKI has a high incidence worldwide, and approximately 4.4% to 22.1% of patients develop acute kidney injury (AKI) after receiving CM injections [3,4]. The poor short-term and long-term health outcomes of CI-AKI are still far from satisfactory and include irreversible kidney injury, prolonged hospitalization, higher medical costs and increased mortality [5,6]. In clinical therapy, the intravenous hydration strategy is commonly used to prevent and treat CI-AKI, but it has limited efficacy [7]. Therefore, there is an urgent need to find new strategies for overcoming current challenges in CI-AKI treatment.

Previously, direct cytotoxicity associated with CM-induced apoptosis, the overproduction of reactive oxygen species (ROS) and renal hemodynamics were the potential pathogenic mechanisms of CI-AKI [8]. The use of drugs targeting apoptosis and oxidative stress regulation may be an effective therapeutic strategy [1,8]. Numerous studies have indicated that apoptosis is related to endoplasmic reticulum (ER) stress, which is a cellular stress response to the accumulation of unfolded or misfolded proteins in the ER lumen [9,10]. ER stress is thought to be an essential pathological process leading to tubular cell injury in AKI, the progression of chronic kidney disease (CKD) and the transition of AKI to CKD [9,11]. Recently, the involvement of ROS-mediated ER stress has been found in contrast-induced renal tubular cell apoptosis [12]. Therefore, the amelioration of ER stress is critical for the attenuation of CI-AKI.

Apelin-13 is the most widespread and bioactive subtype of the apelin family in humans [13]. As an endogenous physiological regulator with antioxidative, anti-inflammatory and antiapoptotic properties, apelin is becoming a therapeutic target for kidney diseases, including renal ischemia-reperfusion injury (IRI), diabetic nephropathy, and renal fibrosis [14–16]. Studies have also shown that the changes in serum apelin levels in patients with kidney diseases may be partly correlated with disease progression [17]. Interestingly, previous studies reported that ER stress was a critical regulator of apelin-mediated protective effects in ischemic stroke, diabetes and heart failure [15,18,19]. Thus, we proposed that exogenous apelin-13 has potential clinical applications for targeting ER stress, oxidative stress and apoptosis. In this study, we aimed to explore the protective role and mechanism of apelin-13 in CI-AKI through *in vivo* and *in vitro* models.

## 2. Materials and methods

### 2.1. Antibodies and specific reagents

Anti-PERK (3192), anti-p-PERK (3179S), anti-CHOP (2895), anti-caspase-4 (4450), anti-cleaved caspase-3 (Cleaved caspase-3) (9664) and anti-GAPDH (5174) antibodies were obtained from Cell Signaling (Danvers, USA). Anti-CHOP (15204-1-AP), anti-78-kDa glucose-regulated protein (GRP78) (66574-1-Ig), anti-Nuclear respiratory factor 2 (Nrf2) (16396-1-AP), anti-Keap1 (60027-1-Ig), anti-GAPDH (10494-1-AP) and anti-β-actin (20536-1-AP) were obtained from Proteintech (Chicago, USA). Anti-apelin (ab125213) and anti-caspase-12 (ab62484) were obtained from Abcam (Cambridge, UK). Anti-β-tubulin (GB11017) was obtained from Servicebio (Wuhan, China). All secondary antibodies were obtained from Abcam (Cambridge, UK). Apelin-13 (A6469, purity ≥ 95.0%) and 4-phenylbutyrate (4-PBA) (P21005) were purchased from Sigma-Aldrich (St. Louis, USA). GSK2656157 (5.04651) was purchased from EMD Millipore (Massachusetts, USA). Tunicamycin (TM) (ab120296) was purchased from Abcam (Cambridge, UK). Iohexol was purchased from GE Healthcare (Shanghai, China).

### 2.2. Cell culture

Human proximal tubular cell lines (HK-2 cells) were cultured in DMEM/F12 medium containing 10% fetal bovine serum and 1% penicillin-streptomycin (Gibco, Waltham, USA) in a humidified incubator at 37 °C with an atmosphere containing 5% CO_2_. CM injury was induced by adding nonionic low-osmolar contrast media iohexol. Briefly, after being washed with phosphate-buffered saline, HK-2 cells were incubated in the medium containing iohexol (200 mg iodine/mL) for 6 h. Additionally, the cells were treated according to the grouping requirements and collected for further analysis.

### 2.3. Animals and surgical protocol

All animal experiments were reviewed and approved by the Institutional Animal Care and Use Committee of Central South University (NO. 100:2020sydw0899). Sprague-Dawley rats (male, 7 weeks, 220–240 g) were purchased and raised at the Department of Laboratory Animals of Central South University. All rats were housed in the pathogen-free animal facility with free access to food and water under a 12 h light-dark cycle and were acclimatized for 7 days before each animal experiment.

The rats were divided into five groups: control group (*n* = 5), 100 nM/kg apelin-13 group (*n* = 5), iohexol group (*n* = 5), iohexol + 10 nM/kg apelin-13 group (*n* = 5), and iohexol + 100 nM/kg apelin-13 group (*n* = 5). The model of rat CI-AKI was established as previously described [20]. Blood samples were collected from the orbital venous plexus through capillary glass tubes and used to measure serum creatinine (SCr) and blood urea nitrogen (BUN) before the rats were deprived of water. All rats that had been dehydrated for 48 h were injected intraperitoneally with furosemide (10 mL/kg) 30 min prior to the time point at which they were injected with iohexol (15 mL/kg). Apelin-13 (10 nM/kg, 100 nM/kg) was injected 10 min before the iohexol injection. Apelin-13 and iohexol were administered intravenously *via* rapid tail vein injection as previously described. The rats were sacrificed 24 h after the iohexol injection, and kidney tissues and blood samples were collected for further experiments (Figure S1).

**Figure 1.**
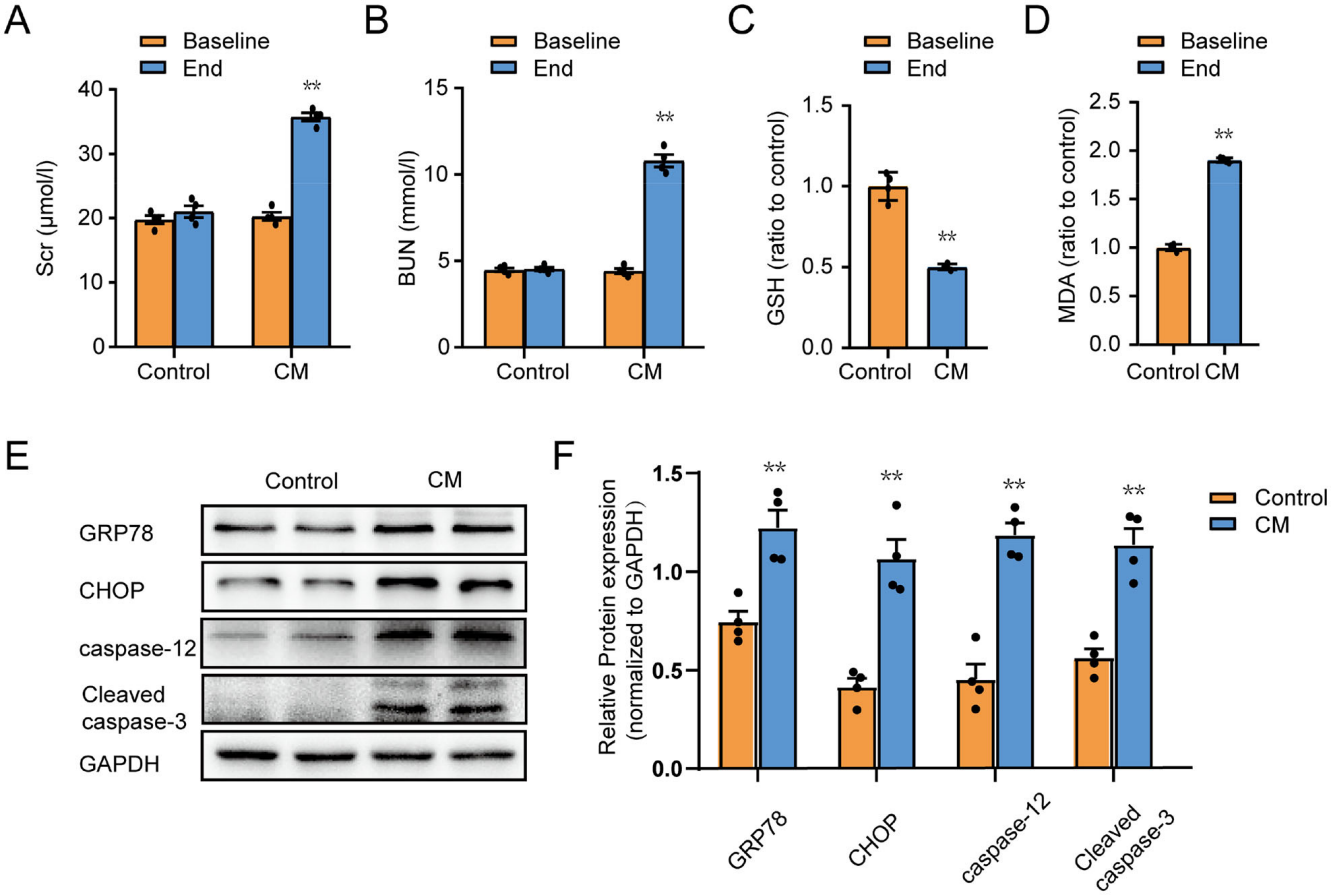
Iohexol induces ER stress, oxidative stress and apoptosis in rat kidney tissues. The method for building the CI-AKI rat model has been described in our previous article. (A, B) Changes in the levels of Scr and BUN. (C) Renal GSH content. (D) Renal MDA activity. (E, F) Representative immunoblot analysis and semi-quantitative analysis of GRP78, CHOP, caspase-12 and Cleaved caspase-3, GAPDH was used as a loading control. Data are expressed as means ± SEMs. *n* = 4. *p < 0.05, **p < 0.01, significantly different from control group.

### 2.4. Cell viability assay

The viability of HK-2 cells was measured with a cell counting kit-8 (CCK-8) (Dojindo Molecular Technologies, Tokyo, Japan) according to the manufacturer’s instructions. Cells were seeded in 96-well plates at a density of 5000 cells/well, and eight replicate wells were used for each group. Then, the cells were incubated with iohexol or apelin-13 for the indicated times. Ten microliters of CCK-8 solution were added to each culture well, followed by incubation for 2 h at 37 °C. A microplate reader (MD SPECTRA M2, Molecular Devices, Sunnyvale, US) was used to spectrophotometrically measure the absorbance at 450 nm. HK-2 cells cultured in DMEM/F12 medium were used as a negative control. Culture media without cells served as blank controls. Cell viability was calculated and expressed as a percentage of the absorbance of the treated group to that of the control group.

### 2.5. Immunoblot analysis

Immunoblot analysis was performed according to standard procedures. A BCA Protein Detection Kit (Beyotime Institute of Biotechnology, Shanghai, China) was used to determine the protein concentrations. The protein samples were electrophoresed on a polyacrylamide gel at suitable concentrations and then transferred to polyvinylidene fluoride membranes. The polyvinylidene fluoride membrane was then blocked with 5% bovine serum albumin or 5% fat-free milk for 1 h, and then incubated overnight with specific primary antibodies diluted according to the manufacturer’s recommendations, followed by incubation with horseradish peroxidase-conjugated secondary antibodies. Antigen-antibody complexes on the membranes were detected using an enhanced chemiluminescence kit (Thermo Fisher Scientific, Rockford, USA). Images were obtained by Tanon 5200 Multi Image Analysis software 1.0 (Tanon, Shanghai, China). Finally, the band intensity was evaluated by ImageJ software.

### 2.6. Transmission electron microscope analysis (TEM)

Fresh samples were fixed in 2.5% glutaraldehyde solution, followed by conventional dehydration, osmosis, embedding, sectioning, and staining as previously described [21]. Typical images were acquired with a Hitachi H7700 electron microscope (Hitachi, Tokyo, Japan).

### 2.7. Determination of blood parameters

SCr and BUN were measured using the automatic biochemical analyzer Hitachi 7170A (Hitachi, Tokyo, Japan) in the laboratory department of Second Xiangya Hospital of Central South University.

### 2.8. Hematoxylin-eosin (HE) staining

Renal tissues were fixed in 4% paraformaldehyde solution, dehydrated, and paraffin-embedded. Paraffin-embedded kidney tissues were sectioned at a thickness of 4 μm and then stained with hematoxylin-eosin for histopathological analysis. Ten high-magnification (× 200) fields in the cortex and outer medulla of HE-stained kidney sections were randomly selected for semiquantitative analysis of morphological alterations. The specimens were scored based on the percentage of damaged renal tubules as previously described: 0, no damage; 1, < 25% damage; 2, 25% − 50% damage; 3, 50% − 75% damage; and 4, > 75% damage.

### 2.9 Renal immunohistochemistry (IHC)

Paraffin-embedded kidney tissues were sectioned at a thickness of 4 μm for IHC analysis. After dewaxing, rehydration, blocking, and antigen retrieval, the kidney tissue sections were exposed to anti-apelin antibodies (1:100) at 4 °C overnight. Then the sections were incubated with biotinylated goat anti-rabbit secondary antibodies (PV-9000, Zhongshan Jinqiao Biotechnology, Beijing, China) for 30 min at room temperature. A DAB kit (ZLI-9018, Zhongshan Jinqiao Biotechnology, Beijing, China) was used to detect the signal of the antigen-antibody complexes. Finally, the slides were counterstained in hematoxylin.

### 2.10 Immunofluorescence (IF) staining

Paraffin-embedded kidney sections were also used for IF studies. The sections were deparaffinized and sequentially incubated with 0.1 M sodium citrate for antigen retrieval, and 3% H_2_O_2_ to block endogenous peroxidase activity. Then 2% normal goat serum blocking buffer was used to reduce nonspecific binding. The sections were then incubated with anti-CHOP (Proteintech, 1:200) and anti-GRP78 antibodies (1:500) at 4 °C. After being incubated overnight incubation at 4 °C, the samples were incubated with secondary antibodies for 1 h at 37 °C in the dark. The sections were counterstained using the antifade mounting medium with DAPI (P0131, Beyotime, Shanghai, China) and then observed and photographed under a fluorescence microscope (BX51, Olympus, Tokyo, Japan).

### 2.11. Malondialdehyde (MDA), glutathione (GSH) and ROS assays

Commercial kits were used to test MDA (A003-1, Jiancheng Bioengineering Institute, Nanjing, China) and GSH (S0052, Beyotime, Shanghai, China) concentrations according to the manufacturer’s instructions.

Dihydroethidium (DHE) staining was used to measure ROS levels in renal tissues as previously described [22]. The whole process can be divided into three parts. First, after the rat kidneys were collected, the kidney was rinsed in cold PBS and then placed in Tissue-Tek optimal cutting temperature compound, snap frozen in liquid nitrogen and stored at -80 °C. Second, freshly isolated 20 μm-thick kidney slices were incubated in 10 μm DHE (Thermo Fisher Scientific, Rockford, USA) for 30 min in the dark at 37 °C and then counterstained with DAPI (P0131, Beyotime, Shanghai, China) at room temperature. Finally, photos were taken with a fluorescence microscope (BX51, Olympus, Tokyo, Japan), and the fluorescence intensity in 10 random optical sections was determined with ImageJ software.

ROS levels in cells were tested by a ROS assay kit purchased from Beyotime Institute of Biotechnology according to the manufacturer’s instructions (S0033S, Shanghai, China). 2′,7′-Dichlorofluorescin diacetate (DCFH-DA) was prepared as a 10 mM solution using a serum-free medium. After being processed according to the grouping requirements, the cells were incubated in a medium containing 10 μM DCFH-DA at 37 °C for 20 min. Finally, fluorescence microscopy (BX51, Olympus, Tokyo, Japan) was used to observe the ROS production in HK-2 cells.

### 2.12. Apoptosis assay

Apoptosis in renal tissues was detected using terminal deoxynucleotidyl transferase dUTP nick-end labeling (TUNEL) reagent (12156792910, Roche Life Science, Basel, Switzerland) according to the manufacturer’s protocol. Briefly, the dewaxed kidney tissue sections were permeabilized with 0.1 M sodium citrate for 30 min. Then, the sections were incubated with TUNEL reaction mixture in the dark at 37 °C for 1 h. The number of TUNEL-positive cells and total cells in different tissue sections were counted in 10 representative fields per section by using fluorescence microscopy (BX51, Olympus, Tokyo, Japan). TUNEL-positive cells are represented as a percentage of total cells.

### 2.13. Statistical analysis

All quantitative data are representative of at least 3 independent experiments. All statistical analyses were performed using SPSS 20.0 software. The results are expressed as the means ± SEMs. A significant difference between the two groups was evaluated using the Student’s *t*-test. A significant difference among three or more groups was determined by one-way analysis of variance (ANOVA) followed by the LSD test for *post hoc* comparisons. *p* < 0.05 was considered significantly different. All statistical results were graphed using GraphPad Prism 6.0 software.

## 3. Results

### 3.1. Iohexol induces ER stress, oxidative stress, and apoptosis in the CI-AKI model

We established a CI-AKI rat model as previously reported to determine whether iohexol induced ER stress in renal tissues *in vivo*. As shown in Figure 1(A–B), both SCr and BUN were significantly increased in rats after iohexol injection (CM group) compared to rats that were injected with saline (control group). The oxidative stress-related indicator GSH was decreased, while MDA was increased in the CM group compared with the control group (Figure 1(C–D)). The protein expression of ER stress indicators (GRP78), ER stress-induced apoptosis indicators (CHOP, caspase-12) and classical apoptosis indicators (Cleaved caspase-3) were all significantly increased compared with the control group (Figure 1(E–F)). These above results demonstrated that iohexol could indeed cause ER stress, oxidative stress and apoptosis in the kidney.

Then we administered iohexol (200 mg iodine/mL) to HK-2 human kidney proximal tubular cells for 0 h, 2 h, 4 h, 6 h, 8 h and 10 h. Iohexol caused a time-dependent decrease in cell viability (Figure S2(A)). Then, we further confirmed that iohexol increased the protein expression of ER stress indicators (GRP78, p-PERK), ER stress-induced apoptosis indicators (CHOP, caspase-4 (an alternative to caspase-4 in humans)) and classical apoptosis indicators (Cleaved caspase-3) in a time-dependent manner by in HK-2 cells immunoblotting (Figure S2(B–H)). TEM also revealed that the rough ER showed swelling, dilatation, and partial vesiculation with many shedding ribosomes in a time-dependent manner of the iohexol groups compared to the control group (Figure S2(I)). Taken together, these results indicate that iohexol triggers the induction of ER stress and apoptosis in renal tubular epithelial cells.

**Figure 2.**
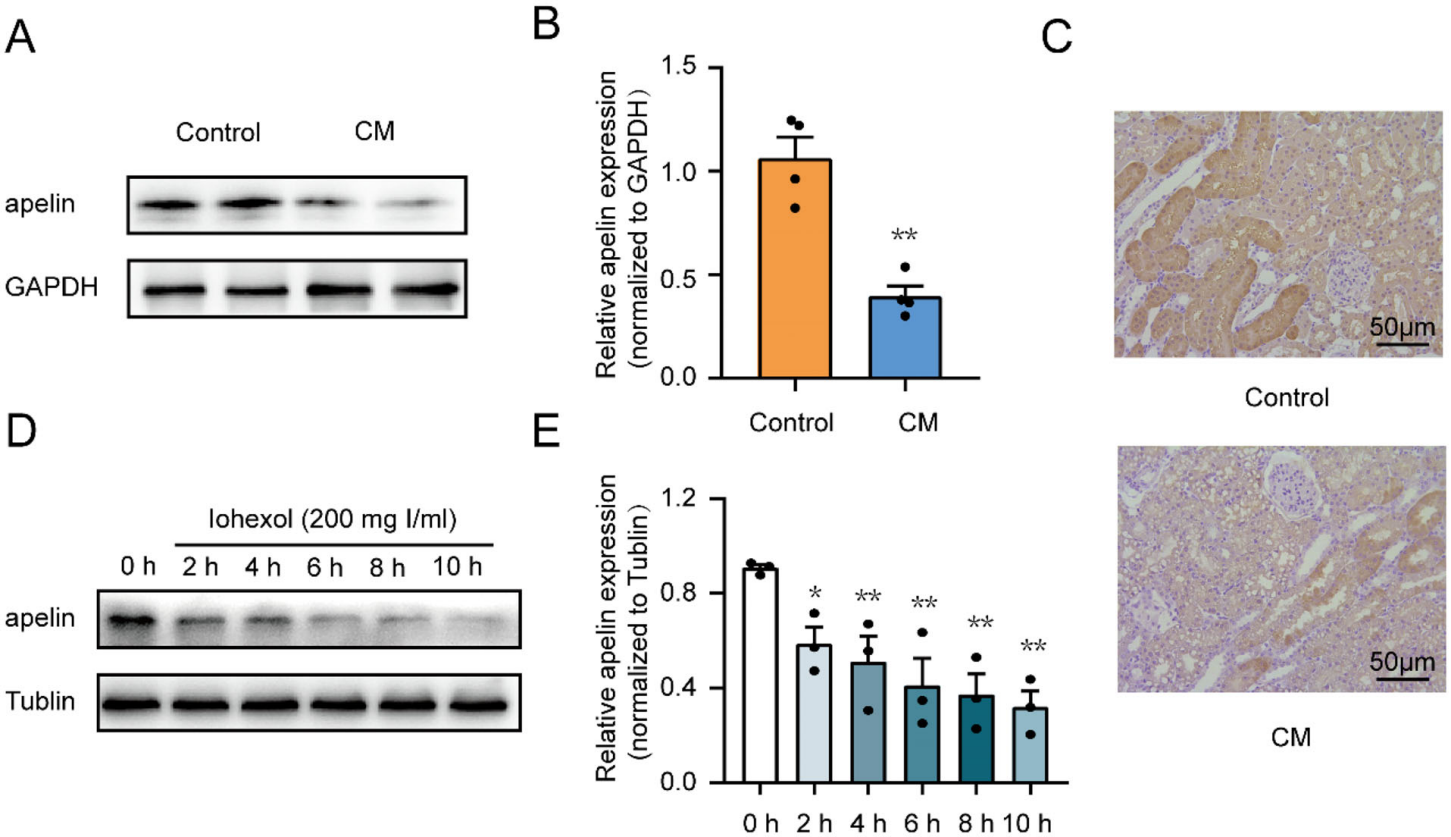
Iohexol intervention reduced apelin expression in tubular epithelial cells in vitro and in vivo. HK-2 cells were treated with iohexol (200 mg iodine/mL) at the indicated time points. The expression of apelin was detected by immunoblot analysis. (A, B) Representative immunoblot analysis and semi-quantitative analysis of apelin in rat kidney tissues, GAPDH was used as a loading control (*n* = 4). (C) Representative immunohistochemical staining of apelin. Scar bar, 50 μm. (D, E) Representative immunoblot analysis and semi-quantitative analysis of apelin in HK-2 cells. Tubulin was used as a loading control (*n* = 3). *p < 0.05, **p < 0.01, significantly different from the control group. Data are expressed as means ± SEMs.

### 3.2. Iohexol reduced apelin expression in rat kidney tissues and HK-2 cells

The effect of the iohexol on apelin expression was examined. In the CI-AKI rat model, immunoblot analysis showed a significant reduction in apelin in kidney tissues after iohexol intervention (Figure 2(A,B)). Immunohistochemical analysis also revealed lower apelin levels in renal tissues after iohexol intervention, and the reduction occurred mainly in renal tubular cells (Figure 2(C)). As shown in Figure 2(D–E), immunoblot analysis showed a sustained decrease in apelin expression with prolonged iohexol treatment in the *in vitro* model. Taken together, these results suggested that apelin was downregulated by iohexol, and that apelin might modulate renal tubular injury.

### 3.3. Apelin-13 attenuated renal injury induced by iohexol *in vivo*

**Figure 3.**
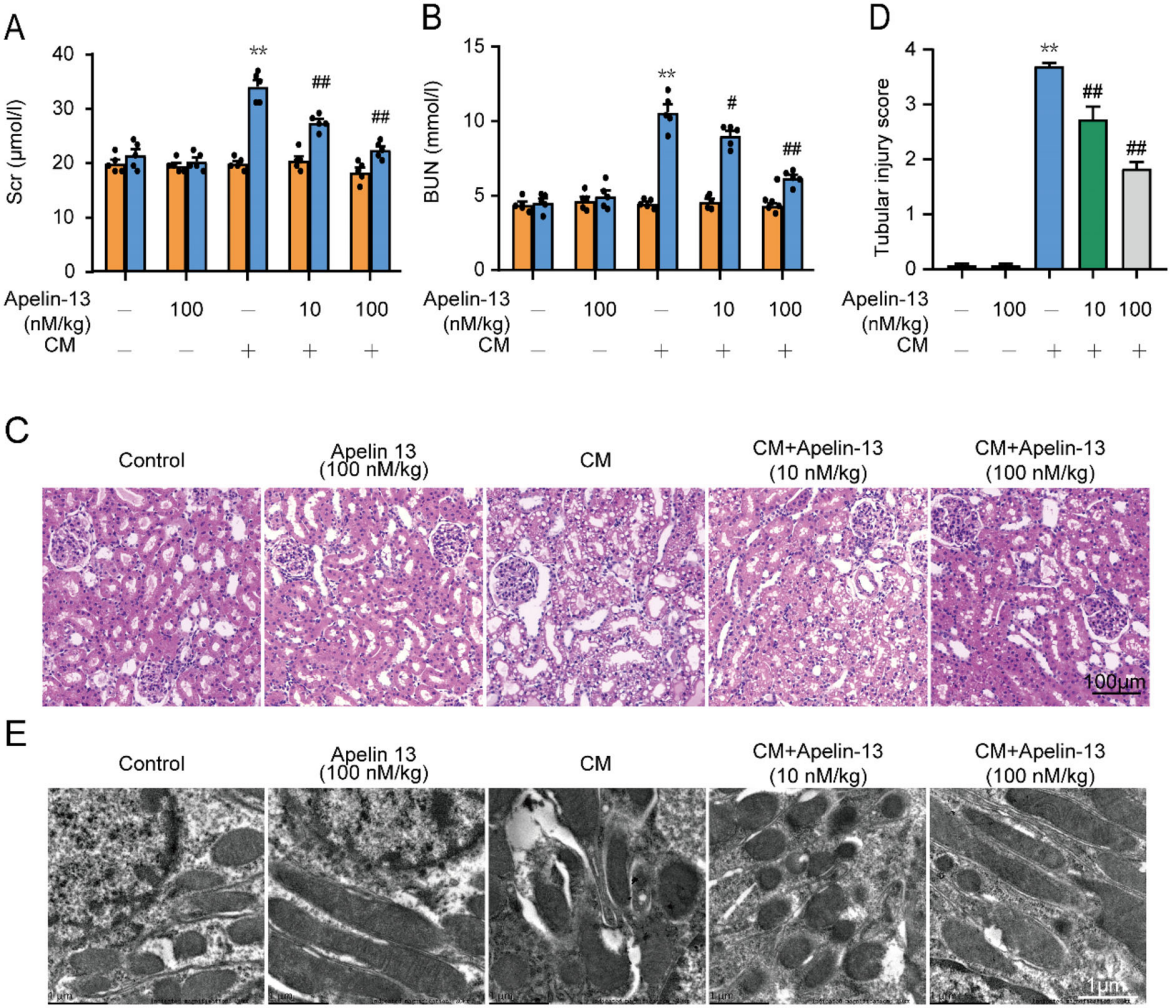
The protective effects of apelin-13 on renal function and pathological injury in rat kidneys. We observed the changes of SCr, BUN, and renal histopathology changes through HE staining in the control group, 100 nM apelin-13 group, iohexol group, iohexol + 10 nM apelin-13 group and iohexol + 100 nM apelin-13 group. (A) Changes of SCr level. (B) Changes of BUN level. (C) Representative images of HE staining. Scar bar, 100 μm. (D) Histopathological score of tubular damage. (E) Representative images of transmission electron microscope analysis (original magnification: ×5000, ×20000; scar bar: 1 μm; under a Hitachi H7700 electron microscope). *p < 0.05, **p < 0.01, significantly different from control group; ^#^*p* < 0.05, ^##^*p* < 0.01, significantly different from the CM group. All quantitative data are expressed as means ± SEMs, *n* = 5.

We further investigated the effect of apelin-13 on a rat CI-AKI model. Compared with the control group, renal function indicators (SCr and BUN) showed no significant difference in the 100 nM apelin-13 group and significant increases in the iohexol group. In contrast, there were significant improvement in SCr and BUN in the CM + 10 nM apelin-13 and CM + 100 nM apelin-13 groups (Figure 3(A,B)). HE staining was used to detect renal histopathology, and the renal tubular injury score of each group was calculated. The vacuolization of renal tubular epithelial cells is an indicator of drug toxicity and a histopathological feature of CI-AKI [8,23]. Almost normal renal tissue structure was observed in the control group and 100 nM apelin-13 groups. Serious tubular epithelial cell vacuolization and shedding, interstitial edema, brush border rarefaction, tubular dilation, and intratubular cast formation were observed in the iohexol group. Apelin-13 treatment significantly alleviated the development of these lesions and tissue damage. Quantitative analysis revealed that following iohexol intervention, apelin-13-treated rats had significantly lower tubular injury scores (∼2.7, ∼1.8) than rats without apelin-13 treatment (∼3.7) (Figure 3(C,D)). In addition, changes in renal tubular epithelial cells were observed by TEM. In the control group, the rough ER was flat-saccular and regularly arranged, with ribosomes attached outside the membrane. Mitochondria and other organelles had normal structures. In the iohexol group, the rough ER showed different degrees of swelling, dilatation, partial vesiculation and ribosomes attached to the ER membrane, and some mitochondria showed swelling, fragmentation, vacuoles, and the loss of cristae. These microstructural alterations in the renal tubular epithelial cells of apelin-13-treated rats were reduced to varying degrees (Figure 3(E)). Collectively, these findings support the therapeutic potential of apelin-13.

### 3.4. The levels of oxidative stress and apoptosis decreased in the renal tissues of CI-AKI rat model after apelin-13 administration

**Figure 4.**
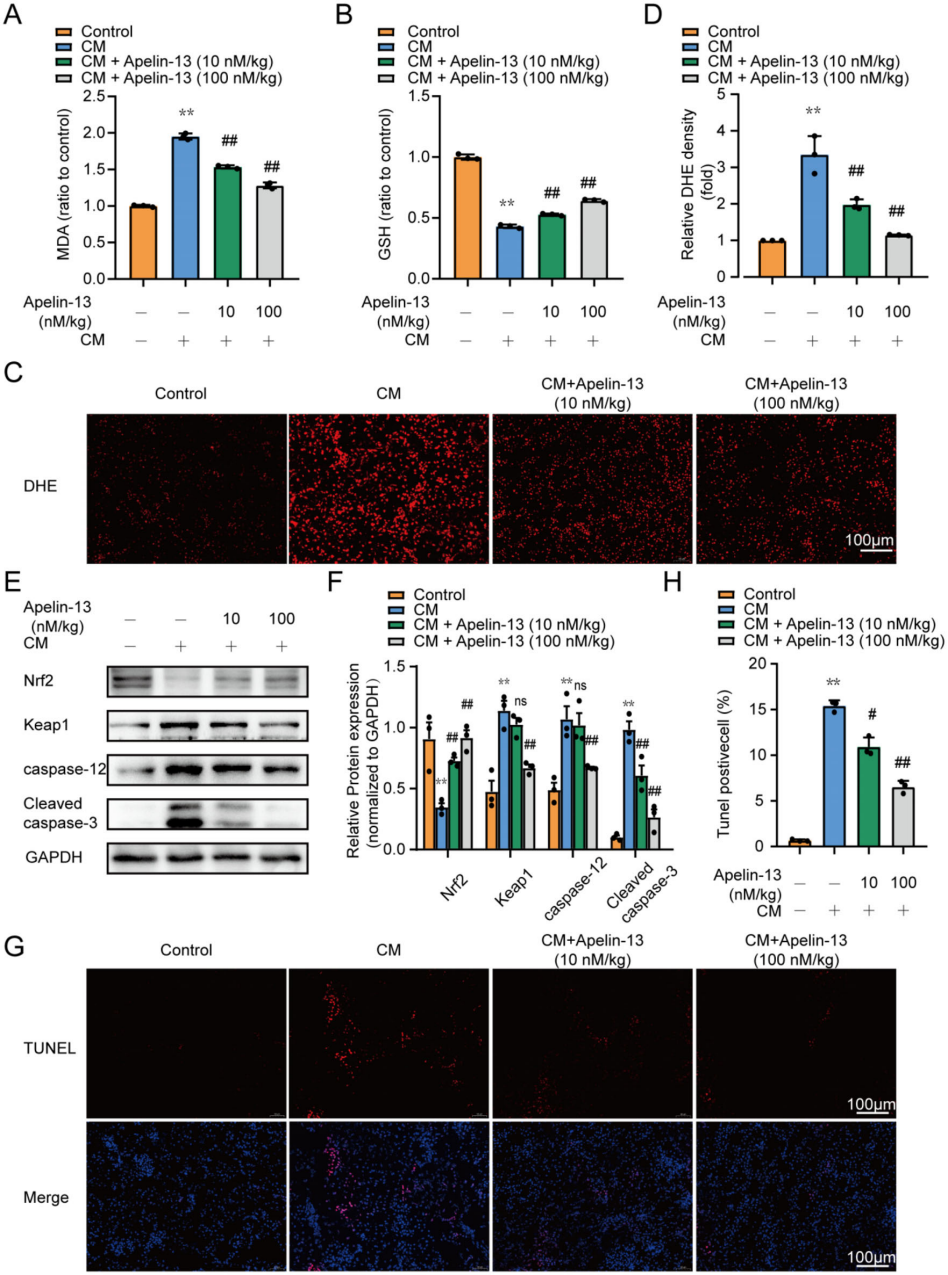
Apellin13 treatment reduced the oxidative stress and apoptosis in rat kidney tissues. Rats were treated as described above. The expression of Keap1, Nrf2 in renal tissues was assessed by immunoblot analysis. Oxidative stress in renal tubular cells was assessed using dihydroethidium (DHE). Apoptosis levels of renal tubular cells were assessed using TUNEL staining. (A) Renal MDA content. (B) Renal GSH activity. (C, D) Representative images of DHE staining and semi-quantitative analysis of DHE fluorescence intensity, Scar bar, 100 μm. (E, F) Representative immunoblot analysis and semi-quantitative analysis of Keap1, Nrf2, caspase-12 and Cleaved caspase-3 in rat kidney tissues, GAPDH was used as a loading control. (G) Representative images of TUNEL staining. The apoptotic cells were detected by TUNEL (red), and the nuclei were detected by DAPI (blue). Scar bar, 100 μm. (H) Quantitative analysis of TUNEL staining positive cells. *p < 0.05, **p < 0.01, significantly different from control group; ^#^*p* < 0.05, ^##^*p* < 0.01, significantly different from the CM group. All quantitative data are expressed as means ± SEMs, *n* = 3.

*In vivo*, we measured MDA and GSH levels in renal tissues. Iohexol increased GSH expression and decreased MDA expression, while apelin-13 treatment ameliorated these changes (Figure 4(A,B)). Moreover, DHE assays revealed a significant increase in oxidative stress in the renal tubular cells of CI-AKI rats, whereas apelin-13 treatment significantly reversed these changes (Figure 4(C,D)). Moreover, since the Keap1-Nrf2 axis serves as a classical antioxidant pathway [21,24], we examined the Keap1 and Nrf2 expression levels by immunoblot analysis. Nrf2 expression was significantly decreased while Keap1 expression was increased in the renal tissues of the iohexol group compared with the control group. The level of Nrf2 activity in the apelin-13-treated group was significantly higher than that in the iohexol group, suggesting that apelin-13 treatment could reduce oxidative stress induced by iohexol in rat renal tissues. Moreover, the results of immunoblot analysis of caspase-12, and Cleaved caspase-3 revealed that apelin-13 reduced iohexol-induced apoptosis (Figure 4(E,F)). TUNEL staining also suggested a decrease in DNA double-strand breaks in the apelin-13-treated group, which was a positive indication of apoptosis (Figure 4(G,H)). These results suggested that apelin-13 plays a vital role in reducing renal tubular cell oxidative stress and apoptosis during CM treatment.

### 3.5. ER stress induced by iohexol was downregulated in rats injected with apelin-13

**Figure 5.**
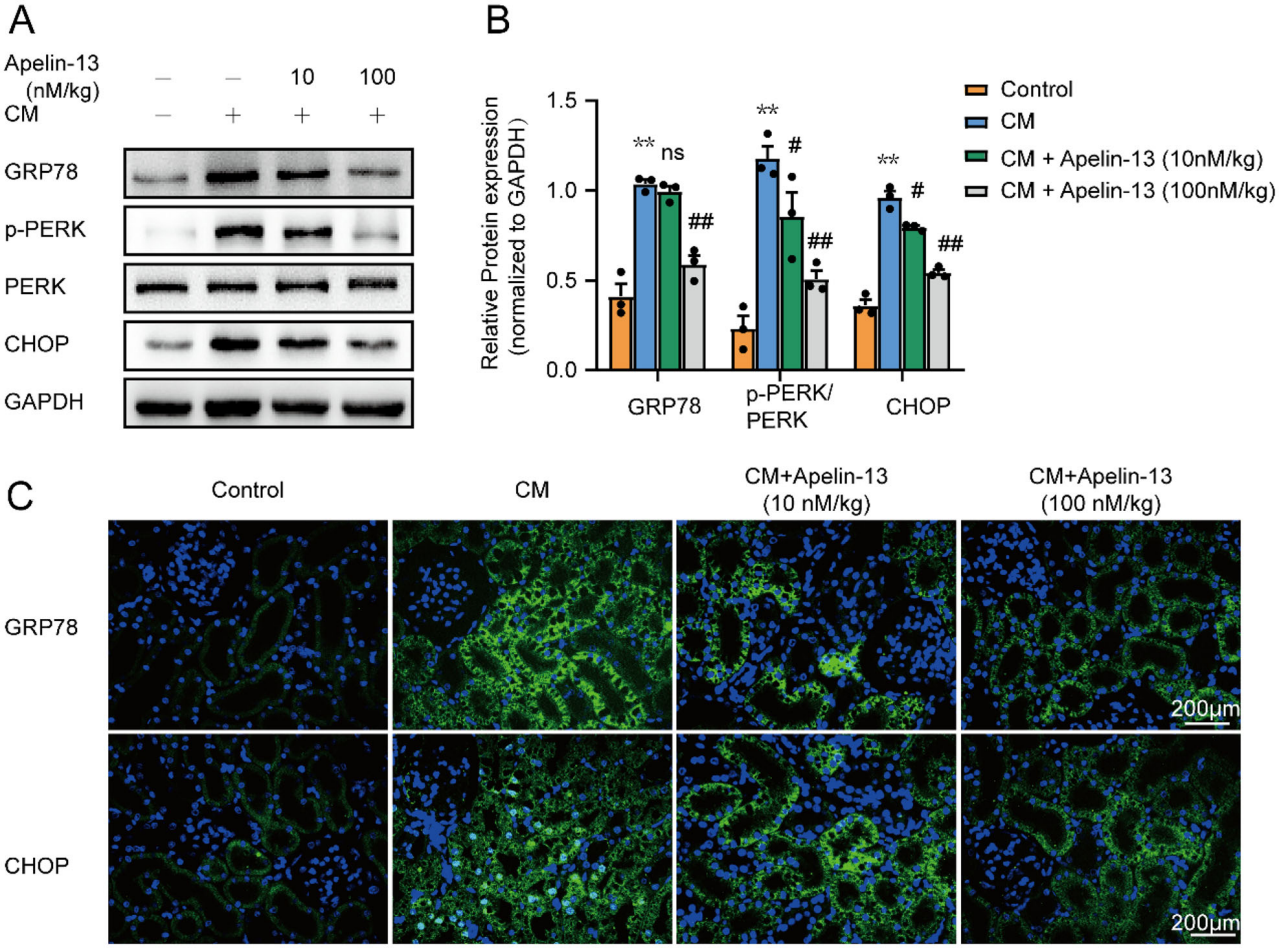
Apelin-13 attenuated iohexol-induced ER stress in rat tubular cells. Rats were treated as described above. The expression of GRP78, p-PERK, PERK and CHOP in kidney tissues was detected by immunoblot analysis. Immunofluorescence was used to detect the expression of GRP78 and CHOP in kidney tissues. (A, B) Representative immunoblot analysis and semi-quantitative analysis of GRP78, p-PERK, PERK, CHOP in rat kidney tissues, GAPDH was used as a loading control. (C) Representative immunofluorescence images of the GRP78 and CHOP. *p < 0.05, **p < 0.01, significantly different from control group; ^#^*p* < 0.05, ^##^*p* < 0.01, significantly different from the CM group. All quantitative data are expressed as means ± SEMs, *n* = 3.

To further confirm the renoprotective effect of apelin-13 on CI-AKI, we examined ER stress by evaluating the expression of ER stress-related proteins. Immunoblot analysis showed that GRP78 and CHOP expression levels and PERK phosphorylation levels were significantly increased in iohexol-treated rat kidney tissues, and these effects were inhibited by apelin-13 treatment (Figure 5(A,B)). The immunoblot analysis results were further validated by the immunohistochemical staining assay, which revealed that GRP78 expression was almost absent in the cortex in the control group, and high levels of GRP78 were observed in the cytoplasm of renal tubules in the iohexol group. Similarly, CHOP expression in the kidney cortex was low in the control group but was increased in the cytoplasm and nucleus after iohexol intervention. The shuttling of CHOP to the nucleus is an indication of the transcriptional activation of CHOP. Notably, the apelin-13 intervention alleviated the increase in the expression of GRP78 and CHOP, especially the accumulation of CHOP in the nucleus (Figure 5(C)). Taken together, these results suggest that apelin-13 may alleviate ER stress in the CI-AKI rat model, further supporting the therapeutic potential of apelin-13.

### 3.6. Apelin-13 protects HK-2 cells from iohexol-induced ER stress, oxidative stress and apoptosis *in vitro*

**Figure 6.**
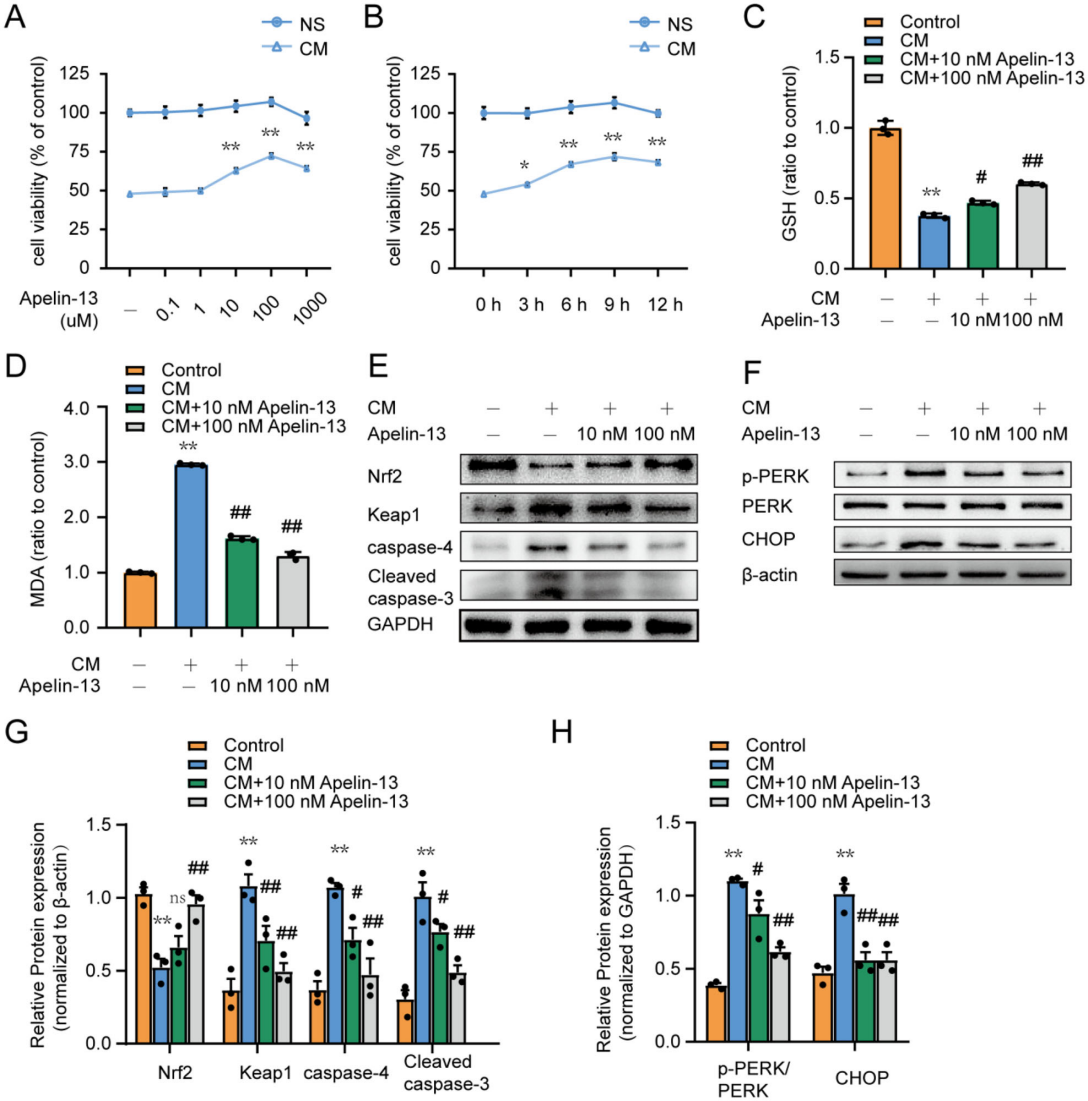
Apelin-13 attenuates iohexol-induced ER stress, oxidative stress and apoptosis in HK-2 cells. (A, B) HK-2 cells were incubated in medium containing 200 mg iodine/mL iohexol and / or interfered with different concentrations of apelin-13 (0, 0.1, 1, 10, 100, 1000 nM) for 6 h. HK-2 cells were incubated in the medium containing 200 mg iodine/mL iohexol for 6 h and / or given the intervention of 100 nM apelin-13 at indicated time points (0 h, 3 h, 6 h, 9 h, 12 h) prior to cell collection. The cells cultured in a normal medium without iohexol were used as a control. Cell viability was detected with CCK-8 assay. Cell viability of the control group was set to 100 %, and other groups were normalized to indicate cell viability changes with the control group (*n* = 8). (C - H) HK 2 cells were incubated in medium containing 200 mg iodine/mL iohexol and apelin(10 nM, 1000 nM) for 6 h. Cells cultured in the normal medium without iohexol or apelin-13 were used as a control. (C) Cell GSH content. (D) Cell MDA activity. (E - H) Representative bands of p-PERK, CHOP, caspase-4, Cleaved caspase-3, Keap1, Nrf2 and semi-quantitative analysis of these protein expression. *p < 0.05, **p < 0.01, significantly different from control group; ^#^*p* < 0.05, ^##^*p* < 0.01, significantly different from the CM group. Data are expressed as means ± SEMs.

We also performed *in vitro* experiments to determine the effect of apelin-13 on ER stress, oxidative stress and apoptosis in HK-2 cells. We initially evaluated the effect of exogenous apelin-13 on the viability of HK-2 cells treated with iohexol (CM, 200 mg iodine/mL, 6 h) by CCK8 assay. As shown in Figure 6(A–B), within a specific range of concentrations and times, apelin-13 intervention dose-dependently improved cell activity. Moreover, the effect was better when apelin-13 was added prior to iohexol or simultaneously. To further confirm whether exogenous apelin-13 could protect renal tubular cells from ER stress through antioxidative and antiapoptotic effects, we added exogenous apelin-13 to HK-2 cells along with iohexol. The MDA and GSH levels in HK-2 cells were consistent with the results of the rat experiments (Figure 6(C,D)). As shown in Figure 6(E,G), Nrf2 was decreased in the iohexol group but increased after apelin-13 treatment. The change in Keap1 was opposite to that of Nrf2. Furthermore, exogenous apelin-13 decreased the expression of caspase-4, Cleaved caspase-3, CHOP and PERK phosphorylation induced by iohexol treatment (Figure 6(E–H)). Apparently, apelin-13 alleviated ER stress, oxidative stress and apoptosis induced by iohexol treatment in HK-2 cells in a dose-dependent manner. These results further validated the *in vitro* experimental results, which indicated the therapeutic potential of exogenous apelin-13 in CI-AKI.

### 3.7. ER stress plays a significant role in the antioxidative and antiapoptotic effects of apelin-13 in HK-2 cells treated with iohexol

To further determine the role of ER stress in iohexol-induced oxidative stress and apoptosis in HK-2 cells, we pretreated HK-2 cells with GSK2656157 and 4-PBA. GSK2656157 is a PERK-specific inhibitor, and 4-PBA is a chemical chaperone that can eliminate the accumulation of unfolded proteins in the ER and attenuate ER stress [25]. As shown in Figures S2 and S3, HK-2 cells were incubated in a medium with 1.0 μM GSK2656157 for 6.5 h, 2.0 mM 4-PBA for 6.5 h and/or 200 mg iodine/mL iohexol for 6 h. The effect of 4-PBA on cell death was evaluated by CCK8 and immunoblot analysis of Cleaved caspase-3. HK-2 cell death induced by iohexol treatment was significantly inhibited by 4-PBA (Figure S3(A)). In contrast to the effect of iohexol alone, the level of MDA was decreased, and the level of GSH was increased after treatment with 4-PBA (Figure S3(B,C)). In addition, 4-PBA reduced the intensity of red fluorescent ROS staining after iohexol treatment (Figure S3(D)). Moreover, immunoblot analysis of Cleaved caspase-3 also showed apoptosis in HK-2 cells induced by iohexol treatment was significantly inhibited by 4-PBA (Figure S3(E–F)). GSK2656157 exerted similar therapeutic effects as 4-PBA (Figure S4(A–F)). Taken together, these results suggest that inhibiting ER stress may provide a promising strategy for the prevention of CI-AKI. However, the effect and detailed mechanism of apelin-13 on ER stress in CI-AKI remain unclear.

**Figure 7.**
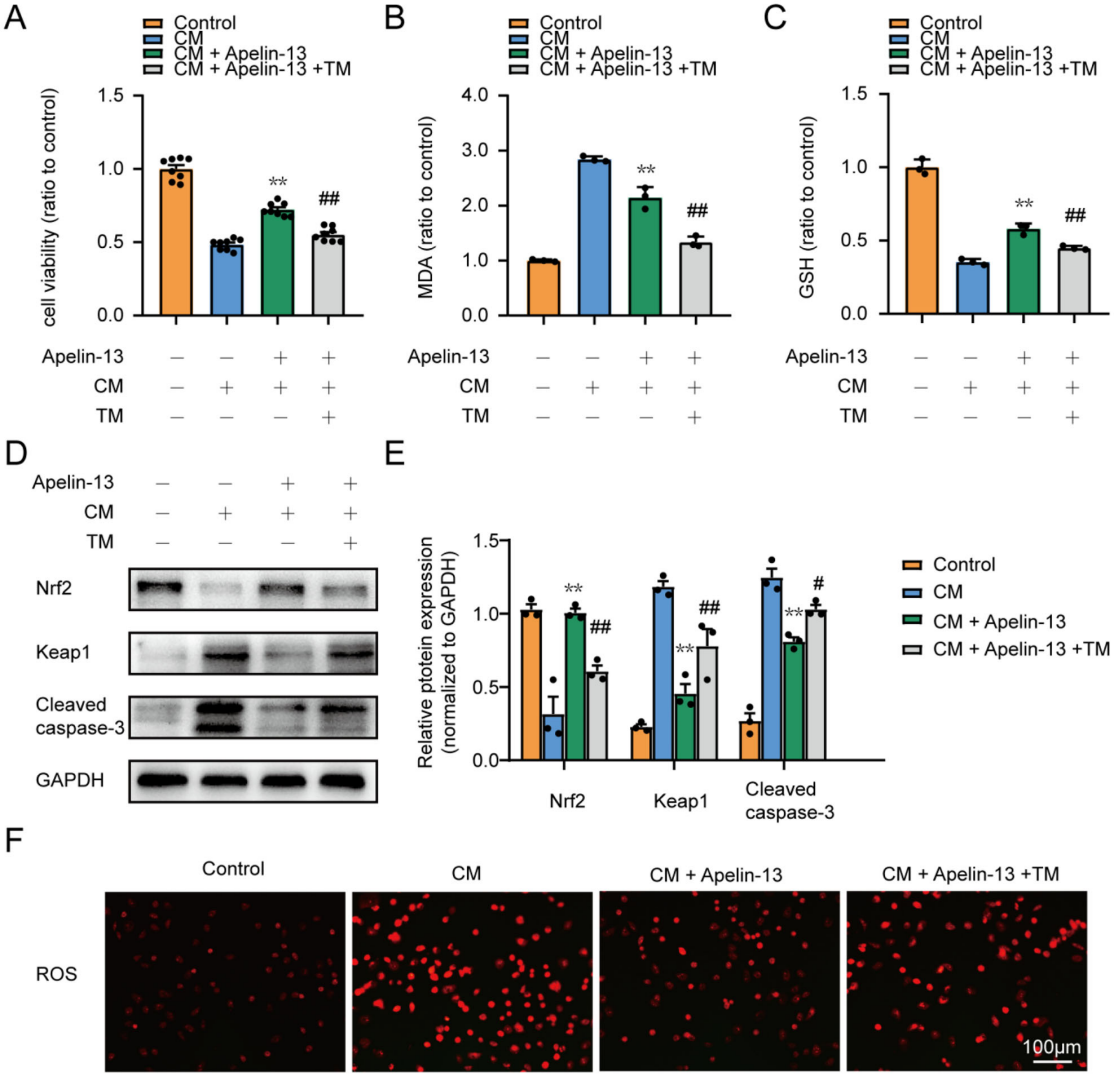
Activation of ER stress abolished the antioxidative and antiapoptotic effects of apelin-13 in HK-2 cells treated with iohexol. HK-2 cells were treated with iohexol (200 mg iodine/mL) for 6 h, apelin-13 (100 nM) for 6 h, and / or TM (100 nM) for 8 h. (A) Cell viability. (*n* = 8). (B) Cell MDA content. (C) Cell GSH activity. (D, E) Representative immunoblot analysis and semi quantitative analysis of Keap1, Nrf2 and Cleaved caspase-3 in HK-2 cells, GAPDH was used as a loading control. (F) Representative images of ROS staining, Scar bar, 100 μm. *p < 0.05, **p < 0.01, significantly different from CM group; ^#^*p* < 0.05, ^##^*p* < 0.01, significantly different from the CM + Apelin-13 group. All quantitative data are expressed as means ± SEMs, *n* = 3.

Based on these results, we presumed that the antioxidative and antiapoptotic effects of apelin-13 on CI-AKI might be related to the inhibition of ER stress. To prove this hypothesis, we used TM, a classical ER stress inducer, to perform rescue experiments *in vitro*. As expected, TM partially reversed the protective effect of apelin-13 treatment (Figure 7(A)). As shown in Figure 7(B–F), TM partially blocked the antioxidative and antiapoptotic effects of apelin-13 in the model of CI-AKI, as shown by MDA, GSH, ROS and the expression of Nrf2, Keap1 and Cleaved caspase-3. As mentioned previously, the results showed that apelin-13 could partially protect HK-2 cells from iohexol-induced cytotoxicity mediated by relieving ER stress.

## Discussion

**Figure 8.**
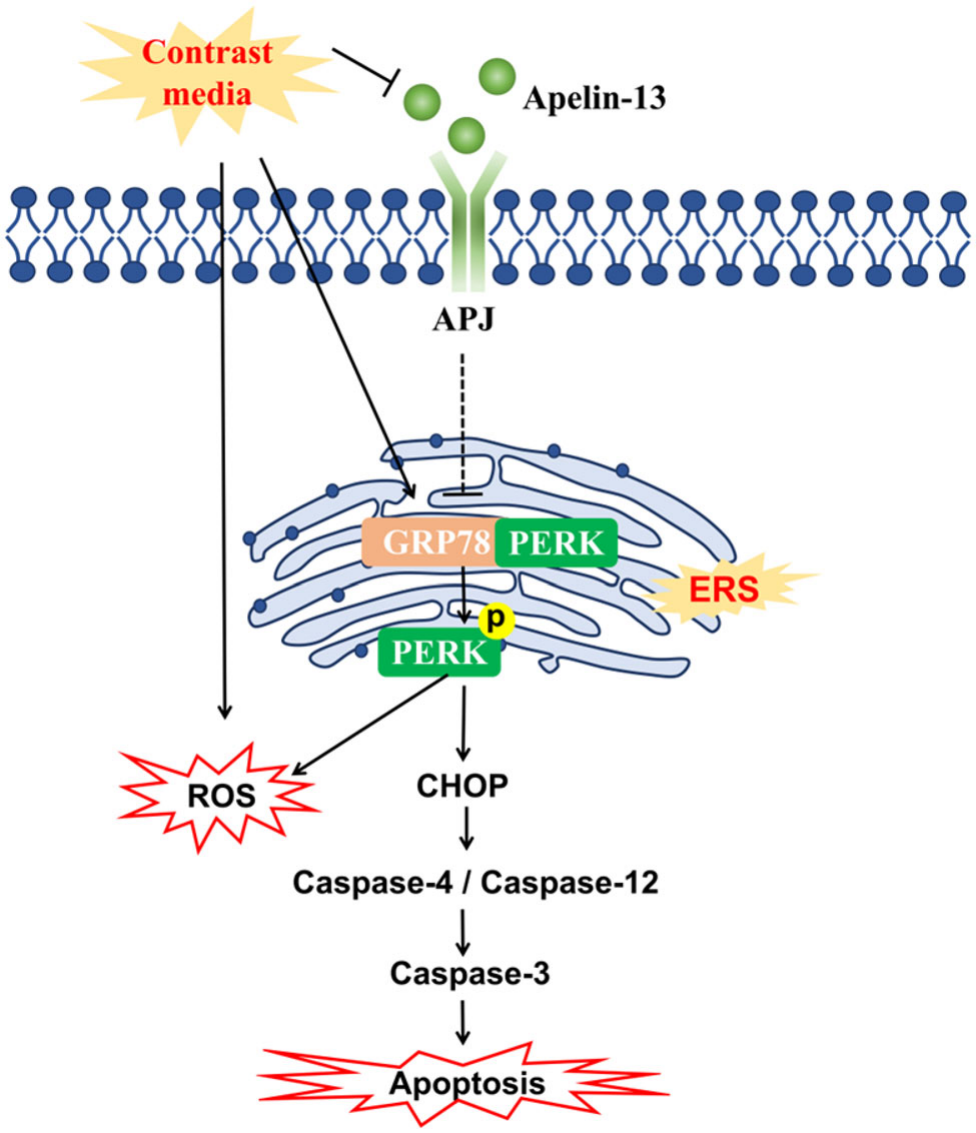
Central illustration - the mechanistic pathway of apelin-13 regulation in CI AKI. ER stress contributed to the oxidative stress and apoptosis of tubular epithelial cells induced by CM. Apelin-13 played a protective role by downregulating ER stress, oxidative stress and apoptosis. Apelin-13 may alleviate oxidative stress and apoptosis through modulating ER stress in tubular epithelial cells.

In the present study, we found that CM downregulated apelin-13 and demonstrated the protective effect of exogenous apelin-13 on CI-AKI rats and CM-treated HK-2 cells. Moreover, our results indicated that exogenous apelin-13 alleviated CI-AKI by reducing ER stress, oxidative stress and apoptosis. Mechanistically, apelin-13 exerted antioxidative and antiapoptotic effects, at least in part, by alleviating ER stress in renal tubular epithelial cells (Figure 8).

CI-AKI is a common cause of AKI and iatrogenic nephropathy, and effective treatments are needed [26]. Impaired renal tubular cells are a hallmark of CI-AKI and are mainly caused by direct cytotoxicity leading to tubular epithelial cell apoptosis, the excessive production of ROS and intrarenal vasoconstriction. Our study and others have shown that renal oxidative stress induced by ROS overproduction results in apoptosis in CI-AKI [21,27], which can be an important pathway that influences kidney damage. In this study, we also examined the oxidative stress and apoptosis indices under the conditions of CI-AKI and showed that apoptosis plays an essential role in the initiation of CI-AKI. Apelin, which is the cognate ligand for the G-protein-coupled receptor APJ, is a member of the adipokine family and has been reported to be a broad regulator of physiology [14]. Apelin was demonstrated to exert protective effects in several renal injury models, such as IRI and diabetic kidney disease, by antioxidative, anti-inflammatory, and antiapoptotic effects [14,19]. However, the pathophysiological effects of apelin in CI-AKI remain elusive. In this study, we provided *in vitro* and *in vivo* evidence that the expression of apelin was reduced during CM intervention. The administration of a certain range of exogenous apelin-13 may alleviate renal tubular epithelial cell injury induced by CM. In addition, apelin-13 treatment attenuated changes in SCr and BUN levels and kidney histological changes in the rat CI-AKI model. More importantly, our study showed the oxidative stress and apoptosis indices under apelin-13 treatment in the rat CI-AKI model, such as the expression of Nrf2, Keap1 and Cleaved caspase-3 and the levels of GSH, MDA ROS and TUNEL, and the downregulation of oxidative stress and apoptosis suggested the antioxidative and antiapoptotic effects of apelin-13 *in vivo* and *in vitro*. Collectively, these findings provide further evidence supporting a potential therapeutic role for apelin-13 in the prevention of CI-AKI.

Apelin-13 has been shown to be beneficial in aortic calcification, ischemic stroke, and subarachnoid hemorrhage by alleviating ER stress [18,28,29]. The protective effect of apelin-13 against ER stress may be mediated by Gαi/Gαq-CK2 signaling [18]. Moreover, increasing evidence suggests that ER stress plays a vital role in renal tubular epithelial injury induced by AKI [9,30]. In terms of the three ER stress pathways, PERK initiates the immediate adaptive reaction to ER stress and crucially determines cell fate in response to ER stress, and so we chose the most classical pathway as the focus of our research. Previous studies confirmed that the PERK-CHOP pathway was altered, and ER stress had an essential effect on apoptosis induced by iodinated CM [12,31–33]. However, injury was mainly induced by high-osmolar contrast media (HOCM), which is currently less frequently used in clinical practice. The relationship between low-osmolar contrast media (LOCM)-induced ER stress and the activation of apoptosis has not been precisely studied. Consistent with LOCM, our study showed that LOCM induced ER stress *in vivo* and *in vitro*. The degree of LOCM-induced cell damage and apoptosis was positively correlated with ER stress levels in a time-dependent manner. By blocking ER stress, we further demonstrated that ER stress was directly involved in contrast-induced tubular epithelial apoptosis. Moreover, apelin-13 treatment reduced ER stress, while the antiapoptotic effect of apelin-13 was weakened after activating ER stress with TM. Therefore, it was proven that the antiapoptotic effect of apelin-13 in CI-AKI was related to inhibiting ER stress.

ER stress and oxidative stress are mutually causative. Low molecular-weight oxidants are produced and amassed in the ER lumen, which is highly oxidative [10,34]. PERK signaling has been reported to be involved in the crosstalk between ER stress and the oxidative stress signaling pathway [35,36]. The Nrf2 pathway is the dominant defense mechanism against oxidative stress [37]. Studies have shown that the Nrf2 pathway is activated in response to ER stress. Activation of the Nrf2 pathway has been shown to reduce the accumulation of misfolded proteins that can contribute to ER stress. In addition, activation of the Nrf2 pathway may also help to reduce inflammation and cell death that can result from ER stress [38,39]. Interestingly, as a regulatory transcription factor, Nrf2 has also been reported to be a direct substrate of PERK [40]. In this study, we provide evidence that the ER stress is associated with oxidative stress induced by CM *via* inhibiting ER stress and specifically inhibiting PERK in CI-AKI, which was shown as a notable change in the levels of MDA, GSH, ROS and Nrf2 expression. Notably, rescue experiments with an ER stress inducer indicated that moderate amounts of apelin-13 prevented iohexol-induced oxidative stress by antagonizing ER stress. Based on these observations, we concluded that apelin-13 maintained ER homeostasis to alleviate contrast-induced renal tubular epithelial injury.

This study has some limitations. First, apelin-13 was administered systemically without tissue or cell-type-specific genetic approaches. Further studies with larger sample sizes are needed to verify the conclusion in other CI-AKI models. Second, apelin-13 may affect renal tubular epithelial cells, renomedullary interstitial cells and renal hemodynamics, but our study focused on the effect of apelin-13 on renal tubular epithelial cell injury *in vitro* experiments. In addition, the critical molecular mechanisms of ER stress in response to apelin-13 treatment and its other precise regulatory mechanisms remain to be investigated in the future.

This study identified the novel beneficial effects of apelin-13 on renal damage in CI-AKI through *in vitro* and *in vivo* experiments. Functionally, treatment with apelin-13 markedly alleviated tubular epithelial damage by reducing ER stress, oxidative stress and apoptosis. Mechanistically, apelin-13 may alleviate oxidative stress and apoptosis by modulating ER stress in tubular epithelial cells. These results suggest that modulation of apelin levels could be regarded as a promising approach to the prevention of CI-AKI in the future.

## Supplementary Material

Supplemental Material

## Data Availability

The data used to support the findings of this study are available from the corresponding authors upon request.
