# Supplementary material for: Apelin-13 alleviates contrast-induced acute kidney injury by inhibiting endoplasmic reticulum stress
Source: Ren Fail. 2023 Feb 22;45(1):2179852. doi: 10.1080/0886022X.2023.2179852 (PMC9970253; doi:10.1080/0886022X.2023.2179852)
Supplement: Supplemental Material [file IRNF_A_2179852_SM6853.pdf]

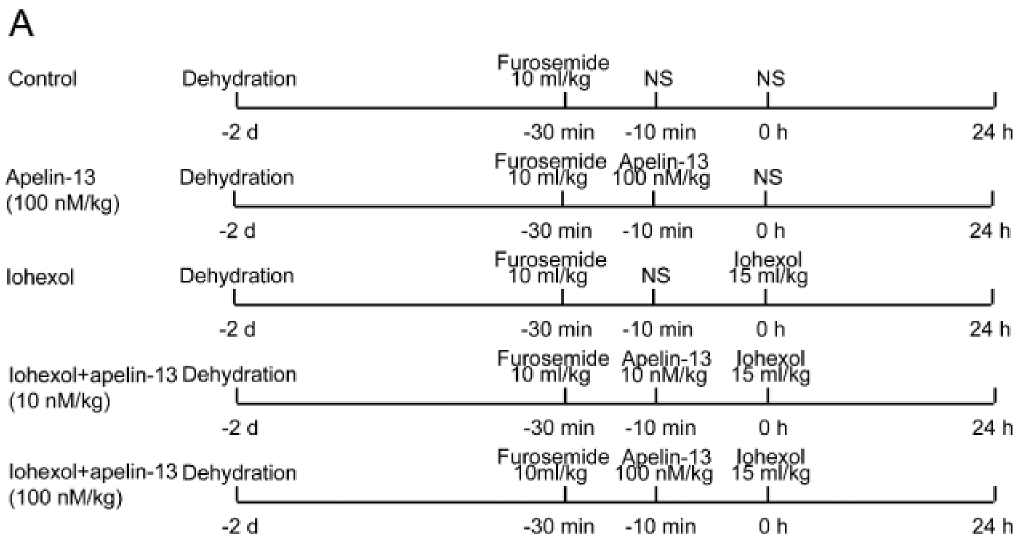

Figure S1. Flow diagrams of animal experiments

Rats intervention experiments. The way to build the CI-AKI rat model was described in our previous article. Sprague-Dawley rats (male, 6 week old) were deprived of water for 2 days before iohexol injection. Blood samples were drawn from the retro-orbital plexus before dehydration. And then, 10 nM/kg apelin-13 or equal volume ratio of normal saline was given by tail vein injection at the time point of 10 min before iohexol injection. Blood samples and kidney tissues were collected 24 h after iohexol injection.

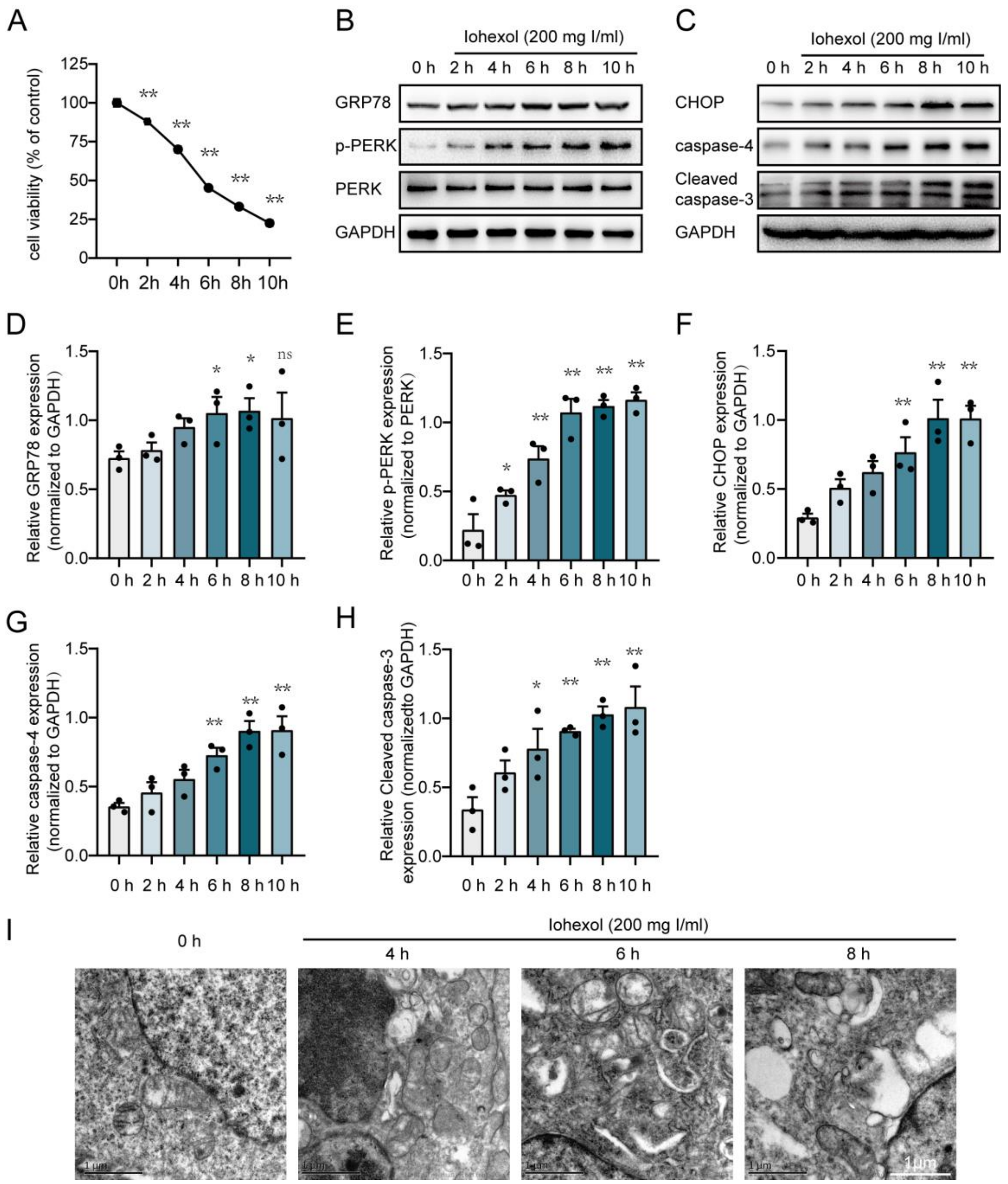

Figure S2. Iohexol induces ER stress and apoptosis in HK-2 cells

HK-2 cells were treated with iohexol (200 mg iodine/mL) at the indicated time points. The expression of GRP78, p-PERK, PERK, CHOP, caspase-4 and Cleaved caspase-3 was detected by immunoblot analysis. (A) Cell viability was detected with CCK-8 assay. Cell viability of the control group (0 h) was set to 100 %, and other groups were normalized to indicate cell viability changes with the control group (n = 8). (B - H) Representative immunoblot analysis and semi-quantitative analysis of GRP78, p-PERK/PERK, CHOP, caspase-4 (an alternative to caspase-12 in human) and

Cleaved caspase-3. GAPDH was used as a loading control (n = 3). (I) Representative micrographs of ER injury in HK-2 cells (original magnifications: ×20000; scar bar: 1 μm; under a Hitachi H7700 electron microscope). * $p < 0.05$, ** $p < 0.01$, significantly different from control group. Data are expressed as means ± SEMs.

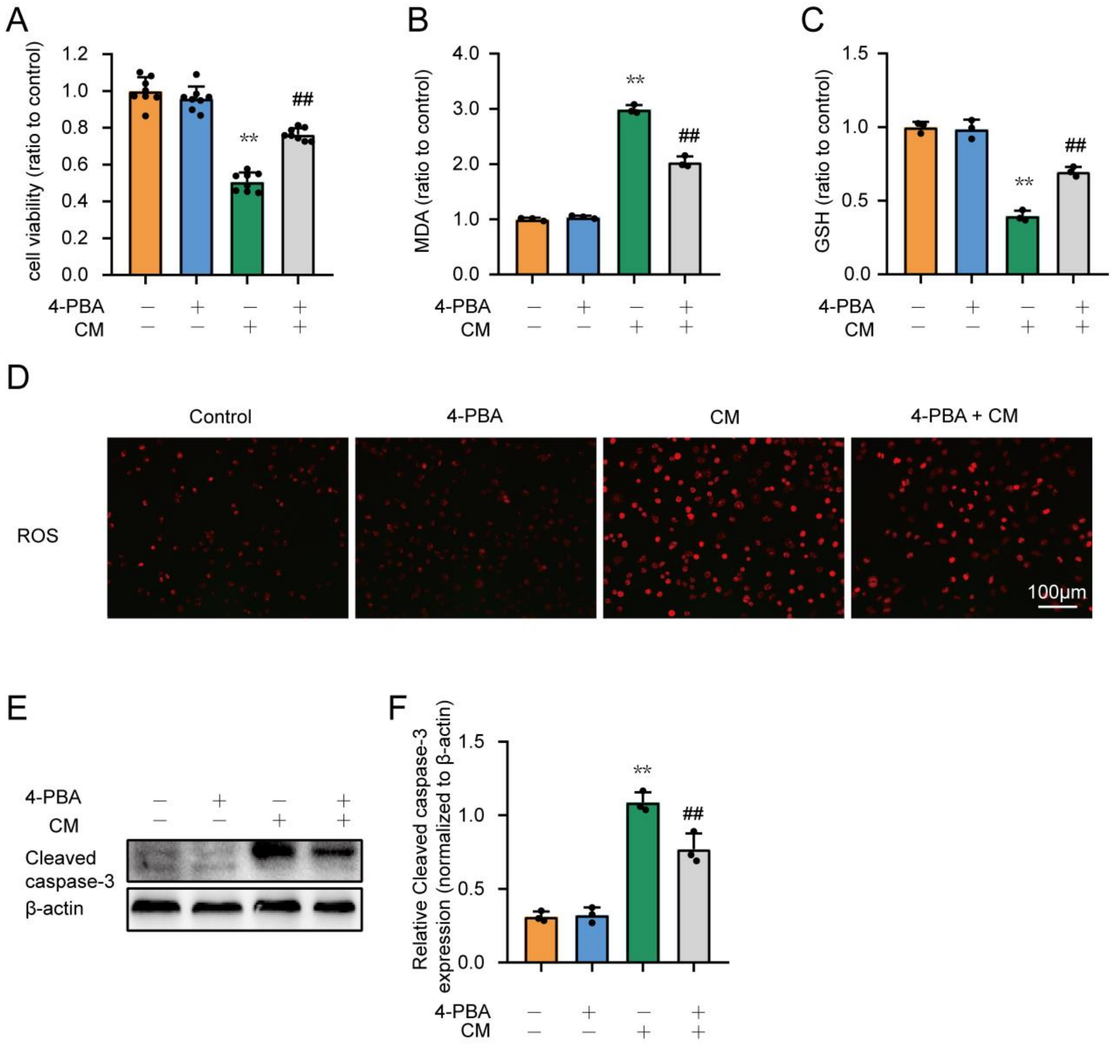

Figure S3. Inhibition of ER stress reduces oxidative stress and apoptosis induced by iohexol in HK-2 cells

HK2 cells were incubated in the medium with 4-PBA at 2.0 mM for 6.5 h or iohexol at 200 mg iodine/mL for 6 h, cell activity was detected by CCK-8 assay and the expression of Cleaved caspase-3 was detected by immunoblot analysis. (A) Cell viability of the control group was set to 100 %, and other groups were normalized to indicate cell viability changes with the control group (n = 8). (B) cell MDA content. (C) Cell GSH activity. (D) Representative images of ROS staining. Scar bar, 100 μm. (E - F) Representative bands of Cleaved caspase-3 and semi-quantitative analysis of protein expression. (n = 3) * $p < 0.05$, ** $p < 0.01$, significantly different from control group; # $p < 0.05$, ## $p < 0.01$, significantly different from the CM group. Data are expressed as means ± SEMs.

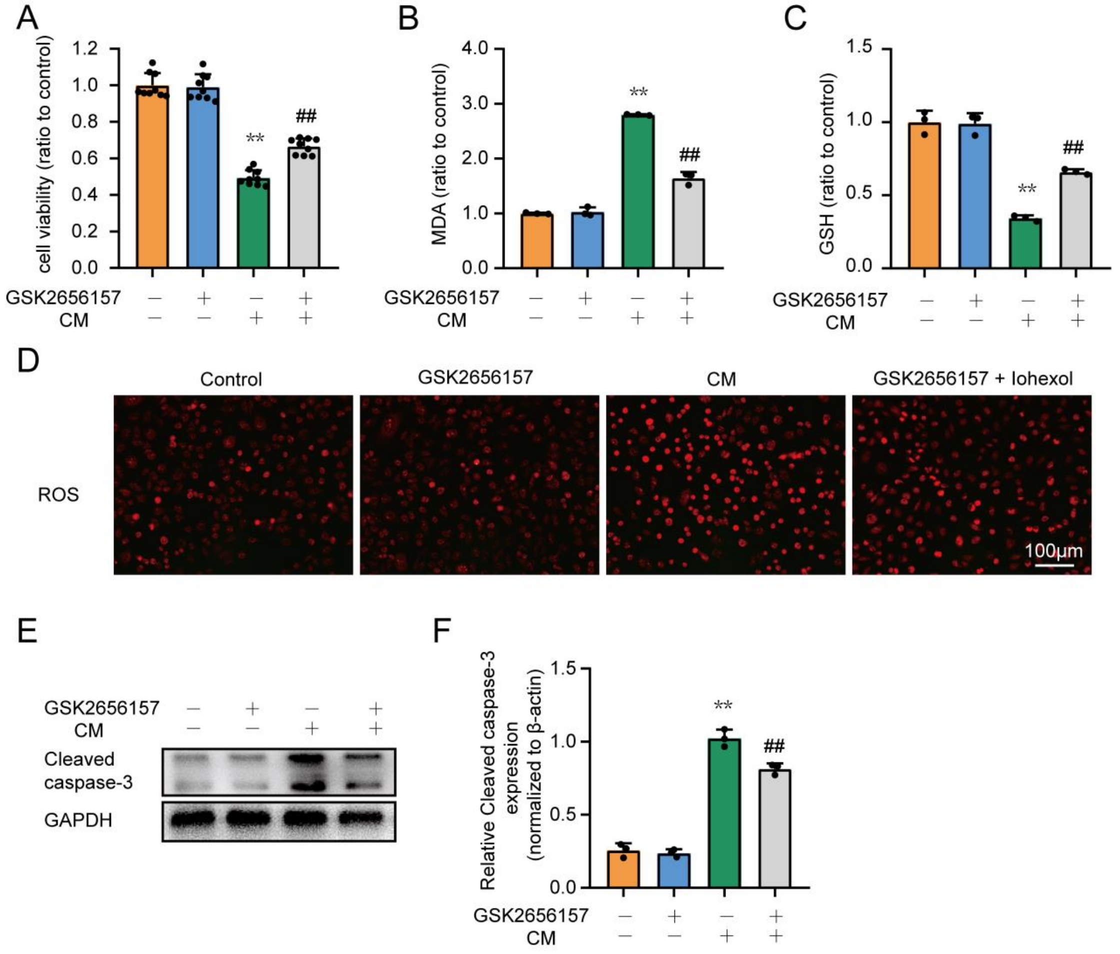

Figure S4. Inhibition of PERK reduces oxidative stress and apoptosis induced by iohexol in HK-2 cells

HK2 cells were incubated in the medium with GSK2656157 at 1.0 μM for 6.5 h or iohexol at 200 mg iodine/mL for 6 h, cell activity was detected by CCK-8 assay and the expression of Cleaved caspase-3 was detected by immunoblot analysis. (A) Cell viability of the control group was set to 100 %, and other groups were normalized to indicate cell viability changes with the control group (n = 8). (B) cell MDA content. (C) Cell GSH activity. (D) Representative images of ROS staining. Scar bar, 100 μm. (E - F) Representative bands of Cleaved caspase-3 and semi-quantitative analysis of protein expression. (n = 3) * $p < 0.05$, ** $p < 0.01$, significantly different from control group; # $p < 0.05$, ## $p < 0.01$, significantly different from the CM group. Data are expressed as means ± SEMs.
